# A comparison of anti-cyclic citrullinated peptides (CCP3 and CCP3.1) autoantibody tests in rheumatoid arthritis

**DOI:** 10.1016/j.plabm.2024.e00420

**Published:** 2024-07-21

**Authors:** Heather A. Nelson, Dipanwita Banerjee, Camille L. Novis, Kevin D. Deane, Marie L. Feser, Vijayalakshmi Nandakumar

**Affiliations:** a ARUP Institute for Clinical and Experimental Pathology, Salt Lake City, UT, USA; b Department of Pathology, University of Utah School of Medicine, Salt Lake City, UT, USA; c University of Colorado School of Medicine Anschutz Medical Campus, Aurora, CO, USA

**Keywords:** Cyclic citrullinated peptide antibodies, Rheumatoid arthritis, Diagnostic performance

## Abstract

**Background:**

Anti-citrullinated protein antibodies (ACPA) are a specific serological biomarker used in the diagnosis of rheumatoid arthritis (RA). In clinical practice ACPA can be identified using immunoassays targeting synthetic cyclic citrullinated peptides (CCP). The 3rd generation anti-CCP IgG antibody (CCP3) offers improved sensitivity compared to the earlier versions. Recently, CCP3.1, capable of detecting both IgG and IgA antibodies, was introduced to enhance sensitivity, especially in patients with early RA.

**Methods:**

We assessed serum CCP3.1 against CCP3 in 331 subjects undergoing RA panel serology, comprising 136 patients with RA and 195 patients without RA. Sera were tested for anti-CCP IgG (CCP3) and anti-CCP IgG/IgA (CCP3.1) antibodies. Clinical performance of these tests was compared at manufacturer-suggested cutoffs. A separate set of 81 patients with a diagnosis of RA by 2010 criteria and whose samples were obtained from within 1-year of RA diagnosis was similarly assessed to evaluate assay performance in an independent clinical RA cohort.

**Results:**

Overall diagnostic accuracy was similar; CCP3 had an area under the curve (AUC) of 0.88, CCP3.1 had an AUC of 0.89. Sensitivity, specificity, positive predictive value (PPV), and negative predictive value (NPV) for CCP3 were 79 %, 91 %, 86 %, and 86 %, respectively. For CCP3.1, sensitivity was 78 %, specificity 93 %, PPV 89 %, NPV 86 %. Both assays demonstrated excellent agreement; positive percent agreement of 94 % and negative percent agreement of 99 %.

**Conclusion:**

Our findings indicate comparable diagnostic accuracy between CCP3 and CCP3.1 assays in these clinical cohorts.

## 1 Introduction

Rheumatoid arthritis (RA) is an autoimmune inflammatory disease [1]. It is a common cause of inflammatory polyarthritis with an estimated global prevalence of 0.1–0.63 % [2]. Diagnosis of RA has been challenged by the heterogenous clinical presentation and course of disease. Modern treatment has shifted toward an aggressive antirheumatic therapy in early disease, creating a need for diagnostic tests highly specific and sensitive for early RA.

Anti-citrullinated protein antibodies (ACPA) have become a leading biomarker for RA diagnosis, with increased sensitivity and specificity compared to other serological markers [3,4]. Citrullination is a posttranslational modification in which arginine is converted to citrulline and occurs naturally during inflammation, apoptosis, and keratinization. ACPA were first discovered in 1995, when it was demonstrated that ACPA was the commonality between perinuclear factor and anti-keratin antibodies detected in patients with RA [5]. Notably these early ACPA were thought to be directed to citrullinated filaggrin, although it is now known that multiple proteins can contain citrullinated proteins and be targeted by autoantibodies. Since their discovery, the role and diagnostic utility of ACPA have been thoroughly investigated, showing a sensitivity of 60–78 % and specificity of 86–99 % for RA.

To improve antigen composition and antibody recognition, a synthetic cyclic citrullinated peptide (CCP) was developed [6]. Since then, several iterations of anti-CCP antibody assays recognizing a mixture of citrullinated peptide antigens have been developed and can be classified into three different generations, all of which show some differences in sensitivity and specificity [[7], [8], [9], [10], [11]]. The first-generation (CCP1) assays have low sensitivity and are no longer widely used [12]. The second generation (CCP2) and third generation (CCP3) assays show improved diagnostic sensitivity and specificity over the CCP1 assay for established RA [13,14].

The CCP3 ELISA detects IgG anti-CCP antibody isotypes and was developed to increase the sensitivity for detection of patients with RA compared to previous generations; however, there is conflicting evidence regarding the comparative diagnostic performance of CCP2 and CCP3. Several published studies suggest that CCP3 does not offer any diagnostic improvement compared to CCP2 [8,[15], [16], [17], [18]]. Yet others have found higher sensitivity of the CCP3 assays compared to CCP2 tests [[19], [20], [21]]. These differences may be attributed to differences in antigens recognized across assays and differences in study populations. Some studies suggest that CCP3 may outperform CCP2 specifically in early RF-negative RA [22,23].

Nevertheless, the third generation CCP assays still only have moderate sensitivity for early disease. Some studies have shown that additional detection of IgA isotype anti-CCP using the same antigens may further improve sensitivity for early RA when used in combination with IgG [19,24]. Thus, a newer assay was developed (CCP3.1) to detect both IgG and IgA anti-CCP antibodies simultaneously [25].

Comparisons of CCP2 and CCP3.1 assays have shown increased sensitivity, but decreased specificity of CCP3.1 compared to CCP2 [26,27], but little is known about the direct comparison between the third generation IgG assays (CCP3) and the IgG/IgA combination assay (CCP3.1). Therefore, the objective of this work is to compare the performance of CCP3.1 and CCP3 assays in two different clinical cohorts.

## 2 Materials & methods

### 2.1 Study samples

Cohort A included specimens from 331 patients that had samples submitted to our laboratory for anti-CCP3 testing as part of a work-up to evaluate for any rheumatic and musculoskeletal diseases. Samples were collected at different time points from symptom onset, ranging from 6 months to more than 2 years. We conducted a retrospective chart review to assess the clinical history and RA diagnosis status of the patients. Among these individuals, 136 had received a diagnosis of RA from our clinicians. This diagnosis was established based on several factors including the presence of pre-existing symptoms lasting more than 6 months, joint involvement, serology results, and findings from inflammatory markers. The remaining 195 participants did not receive a RA diagnosis from our clinicians. Instead, they presented with a spectrum of conditions such as psoriasis, interstitial lung disease, hypothyroidism, diabetes, celiac disease, Raynaud's disease, Sjogren's syndrome, and various other non-specific inflammatory conditions. This subgroup formed our disease control cohort. The demographics of each group are outlined in Table 1. The study was approved by the University of Utah Institutional Review Board (IRB# 00148778).

**Table 1:** Demographics and concentrations of CCP3 and CCP3.1 and across all clinical groups.

Variable	Cohort A - RA patients (n = 136)	Cohort A - Disease Controls (n = 195)	Cohort B (n = 81)
Age (years) Mean (range)	55.7 (24–85)	50.9 (13–84)	51.1 (20–77)
Female sex, n (%)	106 (78 %)	127 (65 %)	55 (68 %)
CCP3, mean units (median; range)	118.5 (145.5; 1–267)	8.4 (4; 1–174)	130.1 (157.0; 3–216)
CCP3.1, mean units (median; range)	110.0 (108.5; 5–237)	11.6 (7.9; 4–211)	137.6 (165.6; 6–230)
CCP3 positive n (%)	107 (79 %)	18 (9 %)	72 (89 %)
CCP3.1 positive n (%)	106 (78 %)	13 (7 %)	74 (91 %)

To further investigate the diagnostic accuracy of CCP3 and CCP3.1 within a distinct clinical setting, we procured 81 samples from a separate cohort (referred to as Cohort B) of individuals from the University of Colorado. All participants in this cohort strictly met the 2010 American College of Rheumatology/European Alliance of Associations for Rheumatology (ACR/EULAR) criteria and had received a diagnosis of RA within 1 year from sample collection, with symptoms persisting for less than 2 years. The use of these samples was approved by the Colorado Multiple Institutional Review Board (IRB# 01–675 and 15–2280).

### 2.2 Measurement of autoantibodies

All samples were tested for CCP3 (QUANTA Lite CCP3 IgG ELISA, Inova Diagnostics, San Diego, CA) and CCP3.1 (QUANTA Lite CCP3.1 IgG/IgA ELISA, Inova Diagnostics, San Diego, CA) by enzyme-linked immunosorbent assay (ELISA) following the procedures recommended by the manufacturer [25,28]. Both assays use the same synthetic cyclic citrullinated peptide (CCP) bound to the surface of a microwell plate. Anti-CCP antibodies in the patient sera will bind to the immobilized CCP, which can then be detected with enzyme-labelled anti-human IgG (CCP3) or an anti-human IgG/IgA conjugate (CCP3.1). Both assay kits are FDA-approved, and the manufacturer suggested cutoff of <20 units was used to classify results in this study (<20 units = negative; ≥20 units = positive). The manufacturer also provides more granular classification of positive results as follows: concentrations 20–39 units are weak positive, 40–59 units are moderate positive, and ≥60 units are strong positives.

### 2.3 Statistical analysis

To evaluate the diagnostic performance of the CCP3 and CCP3.1 assays for the diagnosis of RA, the sensitivity, specificity, positive predictive value (PPV), negative predictive value (NPV), positive likelihood ratio (+LR), negative likelihood ratio (-LR), and odds ratio (OR) of each assay were calculated using the manufacturer's cutoff value. The sensitivity and specificity of CCP3 and CCP3.1 were compared using the Miettinen-Nurminen score confidence interval and a Score Z test [29]. The Cohen kappa coefficient was calculated to measure qualitative agreement between the assays and Spearman's correlation was calculated to compare the quantitative result of each test [30]. Receiver operating characteristic (ROC) curve analysis was used to assess the discriminatory ability of the different tests for RA. The Hanely method was used to compare areas under the receiver operating characteristic curves [31]. Analyses were performed using GraphPad Prism 9 (GraphPad Software, San Diego, California, USA) and Microsoft Excel Analyse-it software (Microsoft Corp, Redmond, Washington).

## 3 Results

**Fig. 1:**
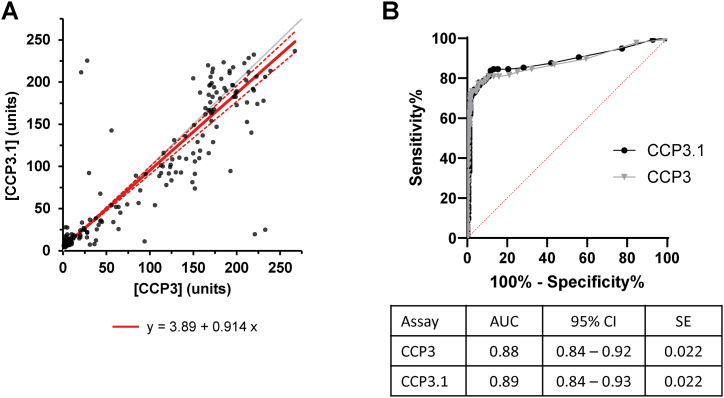
**Comparison of the CCP3 and CCP3.1 assays (n = 331).** (A) Correlation between CCP3 and CCP3.1. Results are displayed in arbitrary units. Gray line corresponds to line of identity. Solid red line corresponds to Passing–Bablok regression and double dotted red line represents 95 % confidence interval bands. Individual test values are displayed as black circles (B) Receiver operating characterisric (ROC) curves for CCP3 and CCP3.1. ROC curves of both assays at manufacturer cut-off value of <20 units.

In cohort A, spearman correlation showed significant positive linearity of results for CCP3 and CCP3.1 (*r*_*s*_ = 0.867, n = 331, p < 0.0001). Passing-Bablok regression yielded a slope of 0.91 (95 % CI, 0.86–0.96) and intercept of 3.89 (95 % CI, 3.62–4.20) (Fig. 1A).

Based on the manufacturers cutoff value of 20 units, CCP3 was positive in 107 of 136 (79 %) patients with RA and 18 of 195 disease controls (9 %). Similarly, CCP3.1 was positive in 106 of 136 (78 %) patients with RA and 13 of 195 disease controls (7 %) (Table 1). Overall qualitative agreement was 97.0 % (Table 2). Cohen's kappa index for categorized values was κ = 0.94, indicating near perfect agreement. Of the 10 discordant results, 8 were weak positives (<39 units), with results near the clinical cutoff (Table 3). Only two patients with discordant results had a strong positive (>60 units) result, which would give an extra point towards diagnosis based on the 2010 diagnostic guidelines. Within the discrepant results, 8 of 10 were positive for CCP3 while negative for CCP3.1; of which, two patients had a clinical RA diagnosis. The other 2 discordances were positive for CCP3.1 while negative for CCP3 and included one clinical RA diagnosis (Table 3).

**Table 2:** CCP3 and CCP3.1 status in cohort A. Discordant results are shaded gray.

		CCP3.1	
		Negative (<20 units)	Positive (≥20 units)
**CCP3**	Negative (<20 units)	204	2
	Positive (≥20 units)	8	117

**Table 3:** Characteristics of samples discrepant for CCP3 and CCP3.1 in cohort A.

Sample	Age (years)	CCP3 (units)	CCP3.1 (units)	RA diagnosis
1	65	221	19	Yes
2	76	20	18	No
3	70	20	16	No
4	65	28	15	Yes
5	38	38	12	No
6	69	94	11	No
7	70	36	8	No
8	24	31	7	No
9	75	3	28	Yes
10	79	5	27	No

Clinical sensitivity, specificity, PPV, NPV, and LRs were calculated for CCP3 and CCP3.1 at the manufacturers’ cutoffs using clinically defined samples (n = 331) (Table 4). CCP3 and CCP3.1 test sensitivity and specificity was equal (sensitivity Z statistic = −0.58, p = 0.56; specificity Z statistic = 1.89, p = 0.59). PPV and NPV were similar for both assays. A marginal increase in LR+ and OR was observed for CCP3.1 compared to CCP3 (LR+ 11.7 vs 8.5; OR 49.5 vs 36.3; CCP3.1 vs CCP3, respectively). ROC analysis demonstrated areas under the curves (AUC) for CCP3 of 0.88 (95 % CI 0.84–0.92) and CCP3.1 of 0.89 (95%CI 0.84–0.93) (Fig. 1B). These AUCs were not statistically different (z = 0.23, p = 0.8), suggesting equivalent diagnostic performance.

**Table 4:** Test performance values for CCP3 and CCP3.1 in cohort A (n = 136 RA patients and 195 disease controls).

	CCP3	CCP3.1
Sensitivity, % (95 % CI)	78.7 (71.1–84.7)	77.9 (70.3–84.1)
Specificity, % (95 % CI)	90.8 (85.9–94.1)	93.3 (88.9–96.1)
PPV, % (95 % CI)	86 (79–90)	89.1 (82.7–93.3)
NPV, % (95 % CI)	86 (82–89)	85.8 (81.5–89.3)
LR+	8.5 (5.5–13.4)	11.7 (7.0–19.9)
LR-	0.24 (0.17–0.32)	0.24 (0.17–0.32)
OR	36.3 (19.3–68.3)	49.5 (24.8–98.5)

Next, we evaluated the clinical utility of CCP3.1 in cohort B, which included patients with a diagnosis of RA (by 2010 ACR/EULAR criteria) within 1 year of sample collection. In this cohort, 72 of 81 (89 %) samples were positive for CCP3 while 74 of 81 (91 %) were positive for CCP3.1 (Table 1). Likewise, overall qualitative agreement was 97.5 % (κ coefficient = 0.86), with the only 2 discordant samples positive for CCP3.1, but negative for CCP3 (Table 5). One of the discordant patients had a strong positive CCP3.1 result (64 units), while the second patient was just weakly positive (25 units).

**Table 5:** CCP3 and CCP3.1 status in cohort B (n = 81 RA patients). Discordant results are shaded gray.

		CCP3.1	
		Negative (<20 units)	Positive (≥20 units)
CCP3	Negative (<20 units)	7	2
	Positive (≥20 units)	0	72

## 4 Discussion

In this study we evaluated and compared the diagnostic performance of two third generation anti-CCP assays for RA; one that measures IgG anti-CCP antibodies only (CCP3) and one that non-discriminately detects both IgG and IgA anti-CCP antibodies (CCP3.1). In our large clinical cohort (cohort A) of patients that had samples submitted for RA serology, we saw no significant difference in diagnostic performance between the CCP3 and CCP3.1 assays. Overall qualitative agreement between CCP3 and CCP3.1 was high at 97 %. There were only ten discordant results, most of which were near the clinical cutoff and would be classified as weak positive by one assay and negative on the other. Furthermore, 7 of the 10 discordances were observed in patients without RA. Indeed, others have shown that using criteria for strong positive as defined by the manufacturer (≥60 units), specificity is greatly increased with only a slight reduction in sensitivity [27]. At this higher cutoff, two cases showed conflicting results in anti-CCP testing: one patient had a strong positive result for anti-CCP IgG (CCP3) but tested negative for anti-CCP IgG/IgA (CCP3.1) and was diagnosed with rheumatoid arthritis (RA), while the other patient did not have RA despite similar discordant findings. Variations in the conjugates used may have obscured specific epitopes, resulting in different antibody reactivity between the two tests. Further evaluation of the second patient with discrepant results could offer more clarity regarding the potential diagnosis of RA. The small number of cases—only 2 out of 331 samples—with significant discrepancies does not provide enough evidence to support the clinical superiority of one assay over the other in our patient cohort. Therefore, based on this data, it is accurate to conclude that CCP3.1 does not provide additional clinical value in our current practice.

The AUC of the ROC curve for the CCP3.1 test was not significantly different from the CCP3 test, suggesting that additional detection of IgA does not improve the diagnostic performance of the third-generation assay. This is consistent with prior reports in which positive IgA anti-CCP results were only observed in patients that were also positive for IgG anti-CCP [32,33]. Likewise, in one of the prior reports demonstrating increased sensitivity of CCP3.1 compared to an CCP2, they showed that this improvement in assay sensitivity was not due to the simultaneous measurement of IgA and IgG, but rather due to a lower clinical cut-off of the assay [27].

Some studies have shown that in addition to IgG, IgA anti-CCP predates the onset of RA by several years [34], leading to the hypothesis that IgA CCP may be particularly useful for diagnosis of early RA. Thus, we also compared CCP3 and CCP3.1 in 81 individuals primarily diagnosed with RA within one year of sample collection and onset of symptoms (cohort B). In this cohort, there was 97.5 % qualitative agreement between both assays. The only two discordant cases were both positive by CCP3.1, but negative by CCP3. One of the positive CCP3.1 results was just weakly positive, but the second was considered a strong positive. This finding lends itself to the possibility that these two patients were only positive for IgA anti-CCP and not IgG, but an independent IgA assay would need to be performed to confirm that.

Our study has several limitations. Cohort B was identified based on a clinical referral system in which RA is preferentially diagnosed based on autoantibody testing and further were selected to meet 2010 ACR/EULAR RA classification criteria. Because anti-CCP is a strong contributor to those criteria, this weights for patients positive for anti-CCP, which may limit our ability to see differences between the CCP3 and CCP3.1 assays in early RA. Nevertheless, we failed to see any notable differences in our routine clinical practice cohort (cohort A), which had a large number of RA patients and appropriate disease controls. Another limitation was both cohorts lacked sufficient patients in early RA with symptom duration of less than 6 months. Lastly, we focused herein on comparison of CCP3 and CCP3.1 assays and did not do direct comparisons to CCP2 assays. Prior work has shown contrasting conclusions in comparing CCP2 and CCP3. In the future, the diagnostic accuracy of CCP2, CCP3 and CCP3.1, as well as other ACPA assays such as anti-mutated citrullinated vimentin (anti-MCV) and different antibody combinations with RF assays (including isotypes IgG, M and A) may be compared.

## 5 Conclusion

Our results have shown that the diagnostic performance of the CCP3.1 assay is equivalent to that of the CCP3 assay in routine clinical practice. There is evidence suggesting that some early RA patients may test positive for IgA anti-CCP even when IgG anti-CCP is negative [34]. However, further studies specifically focusing on IgA anti-CCP are necessary to confirm whether this positivity is indeed attributable to IgA and whether its detection could enhance sensitivity in early RA patient cohorts.

## Funding

Financial support for this work was provided by the 10.13039/100013213ARUP Institute for Clinical and Experimental Pathology at 10.13039/100015079ARUP Laboratories. We are grateful to Dr. Kevin Deane for generously sharing the eighty samples from individuals diagnosed with early RA for inclusion in this study. Kevin Deane and Marie Feser were supported on this project by 10.13039/100000002NIH/10.13039/100000069NIAMS grant P30 AR079369.

## CRediT authorship contribution statement

**Heather A. Nelson:** Writing – review & editing, Writing – original draft, Project administration, Investigation, Formal analysis, Data curation. **Dipanwita Banerjee:** Writing – review & editing, Investigation, Data curation. **Camille L. Novis:** Writing – review & editing, Investigation, Formal analysis, Data curation. **Kevin D. Deane:** Writing – review & editing, Resources, Investigation, Funding acquisition, Data curation, Conceptualization. **Marie L. Feser:** Writing – review & editing, Resources, Data curation. **Vijayalakshmi Nandakumar:** Writing – review & editing, Writing – original draft, Visualization, Resources, Project administration, Investigation, Formal analysis, Data curation, Conceptualization.

## Declaration of competing interest

The authors declare that they have no known competing financial interests or personal relationships that could have appeared to influence the work reported in this paper.

## Data Availability

Data will be made available on request.

## References

[1] Taylor P.C. (2020). Update on the diagnosis and management of early rheumatoid arthritis. Clin. Med..

[2] Finckh A., Gilbert B., Hodkinson B., Bae S.-C., Thomas R., Deane K.D., Alpizar-Rodriguez D., Lauper K. (2022). Global epidemiology of rheumatoid arthritis. Nat. Rev. Rheumatol..

[3] Lee A.N., Beck C.E., Hall M. (2008). Rheumatoid factor and anti-CCP autoantibodies in rheumatoid arthritis: a review. Clin. Lab. Sci..

[4] Chang P.Y., Yang C.T., Cheng C.H., Yu K.H. (2016). Diagnostic performance of anti-cyclic citrullinated peptide and rheumatoid factor in patients with rheumatoid arthritis. Int J Rheum Dis.

[5] Aggarwal R., Liao K., Nair R., Ringold S., Costenbander K.H. (2009). Anti–citrullinated peptide antibody assays and their role in the diagnosis of rheumatoid arthritis. Arthritis Care Res..

[6] Schellekens G.A., Visser H., de Jong B.A., van den Hoogen F.H., Hazes J.M., Breedveld F.C., van Venrooij W.J. (2000). The diagnostic properties of rheumatoid arthritis antibodies recognizing a cyclic citrullinated peptide. Arthritis Rheum..

[7] Coenen D., Verschueren P., Westhovens R., Bossuyt X. (2007). Technical and diagnostic performance of 6 assays for the measurement of citrullinated protein/peptide antibodies in the diagnosis of rheumatoid arthritis. Clin. Chem..

[8] Bizzaro N., Tonutti E., Tozzoli R., Villalta D. (2007). Analytical and diagnostic characteristics of 11 2nd- and 3rd-generation immunoenzymatic methods for the detection of antibodies to citrullinated proteins. Clin. Chem..

[9] Vander Cruyssen B., Nogueira L., Van Praet J., Deforce D., Elewaut D., Serre G., De Keyser F. (2008). Do all anti-citrullinated protein/peptide antibody tests measure the same? Evaluation of discrepancy between anti-citrullinated protein/peptide antibody tests in patients with and without rheumatoid arthritis. Ann. Rheum. Dis..

[10] Gaalen F.A.v., Visser H., Huizinga T.W.J. (2005). A comparison of the diagnostic accuracy and prognostic value of the first and second anti-cyclic citrullinated peptides (CCP1 and CCP2) autoantibody tests for rheumatoid arthritis. Ann. Rheum. Dis..

[11] Whiting P.F., Smidt N., Sterne J.A.C., Harbord R., Burton A., Burke M., Beynon R., Ben-Shlomo Y., Axford J., Dieppe P. (2010). Systematic review: accuracy of anti–citrullinated peptide antibodies for diagnosing rheumatoid arthritis. Ann. Intern. Med..

[12] Bizzaro N., Mazzanti G., Tonutti E., Villalta D., Tozzoli R. (2001). Diagnostic accuracy of the anti-citrulline antibody assay for rheumatoid arthritis. Clin. Chem..

[13] van Gaalen F.A., Visser H., Huizinga T.W.J. (2005). A comparison of the diagnostic accuracy and prognostic value of the first and second anti-cyclic citrullinated peptides (CCP1 and CCP2) autoantibody tests for rheumatoid arthritis. Ann. Rheum. Dis..

[14] Taylor P., Gartemann J., Hsieh J., Creeden J. (2011). A systematic review of serum biomarkers anti-cyclic citrullinated peptide and rheumatoid factor as tests for rheumatoid arthritis. Autoimmune Dis..

[15] Correia M.L., Carvalho S., Fortuna J., Pereira M.H. (2008). Comparison of three anti-CCP antibody tests and rheumatoid factor in RA and control patients. Clin. Rev. Allergy Immunol..

[16] van der Linden M.P., van der Woude D., Ioan-Facsinay A., Levarht E.W., Stoeken-Rijsbergen G., Huizinga T.W., Toes R.E., van der Helm-van Mil A.H. (2009). Value of anti-modified citrullinated vimentin and third-generation anti-cyclic citrullinated peptide compared with second-generation anti-cyclic citrullinated peptide and rheumatoid factor in predicting disease outcome in undifferentiated arthritis and rheumatoid arthritis. Arthritis Rheum..

[17] Zhang W.C., Wu H., Chen W.X. (2014). Meta-analysis: diagnostic accuracy of anti-cyclic citrullinated peptide 2 antibody and anti-cyclic citrullinated peptide 3 antibody in rheumatoid arthritis. Clin. Chem. Lab. Med..

[18] Lutteri L., Malaise M., Chapelle J.P. (2007). Comparison of second- and third-generation anti-cyclic citrullinated peptide antibodies assays for detecting rheumatoid arthritis. Clin. Chim. Acta.

[19] dos Anjos L.M., Pereira I.A., d 'Orsi E., Seaman A.P., Burlingame R.W., Morato E.F. (2009). A comparative study of IgG second- and third-generation anti-cyclic citrullinated peptide (CCP) ELISAs and their combination with IgA third-generation CCP ELISA for the diagnosis of rheumatoid arthritis. Clin. Rheumatol..

[20] Debaugnies F., Servais G., Badot V., Noubouossie D., Willems D., Corazza F. (2013). Anti-cyclic citrullinated peptide antibodies: a comparison of different assays for the diagnosis of rheumatoid arthritis. Scand. J. Rheumatol..

[21] Wu R., Shovman O., Zhang Y., Gilburd B., Zandman-Goddard G., Shoenfeld Y. (2007). Increased prevalence of anti-third generation cyclic citrullinated peptide antibodies in patients with rheumatoid arthritis and CREST syndrome. Clin. Rev. Allergy Immunol..

[22] Jaskowski T.D., Hill H.R., Russo K.L., Lakos G., Szekanecz Z., Teodorescu M. (2010). Relationship between rheumatoid factor isotypes and IgG anti-cyclic citrullinated peptide antibodies. J. Rheumatol..

[23] Swart A., Burlingame R.W., Gürtler I., Mahler M. (2012). Third generation anti-citrullinated peptide antibody assay is a sensitive marker in rheumatoid factor negative rheumatoid arthritis. Clin. Chim. Acta.

[24] Sieghart D., Platzer A., Studenic P., Alasti F., Grundhuber M., Swiniarski S., Horn T., Haslacher H., Blüml S., Smolen J., Steiner G. (2018). Determination of autoantibody isotypes increases the sensitivity of serodiagnostics in rheumatoid arthritis. Front. Immunol..

[25] Quanta Lite CCP3.1 IgG/IgA ELISA [package insert] (2020).

[26] Demoruelle M.K., Parish M.C., Derber L.A., Kolfenbach J.R., Hughes-Austin J.M., Weisman M.H., Gilliland W., Edison J.D., Buckner J.H., Mikuls T.R., O'Dell J.R., Keating R.M., Gregersen P.K., Norris J.M., Holers V.M., Deane K.D. (2013). Performance of anti–cyclic citrullinated peptide assays differs in subjects at increased risk of rheumatoid arthritis and subjects with established disease. Arthritis Rheum..

[27] Sieghart D., Konrad C., Swiniarski S., Haslacher H., Aletaha D., Steiner G. (2022). The diagnostic and prognostic value of IgG and IgA anti-citrullinated protein antibodies in patients with early rheumatoid arthritis. Front. Immunol..

[28] Quanta Lite CCP3 IgG ELISA [package insert] (2020).

[29] Miettinen O., Nurminen M. (1985). Comparative analysis of two rates. Stat. Med..

[30] Landis J.R., Koch G.G. (1977). The measurement of observer agreement for categorical data. Biometrics.

[31] Hanley J.A., McNeil B.J. (1983). A method of comparing the areas under receiver operating characteristic curves derived from the same cases. Radiology.

[32] Svärd A., Kastbom A., Reckner-Olsson A., Skogh T. (2008). Presence and utility of IgA-class antibodies to cyclic citrullinated peptides in early rheumatoid arthritis: the Swedish TIRA project. Arthritis Res. Ther..

[33] Verpoort K.N., Jol-van der Zijde C.M., Papendrecht-van der Voort E.A., Ioan-Facsinay A., Drijfhout J.W., van Tol M.J., Breedveld F.C., Huizinga T.W., Toes R.E. (2006). Isotype distribution of anti-cyclic citrullinated peptide antibodies in undifferentiated arthritis and rheumatoid arthritis reflects an ongoing immune response. Arthritis Rheum..

[34] Kokkonen H., Mullazehi M., Berglin E., Hallmans G., Wadell G., Rönnelid J., Rantapää-Dahlqvist S. (2011). Antibodies of IgG, IgA and IgM isotypes against cyclic citrullinated peptide precede the development of rheumatoid arthritis. Arthritis Res. Ther..

